# Revision laryngeal framework surgery performed by directly pulling the lateral cricoarytenoid muscle

**DOI:** 10.1017/S0022215114001546

**Published:** 2014-08-14

**Authors:** T Kanazawa, D Komazawa, Y Watanabe, K Ichimura

**Affiliations:** 1 Department of Otolaryngology/Head and Neck Surgery, Jichi Medical UniversitySchool of Medicine, Shimotsuke, Japan; 2 Department of Otolaryngology, Shinn-Oyama City Hospital, Oyama, Japan; 3 Tokyo Voice Centre, International University of Health and Welfare, Tokyo, Japan

**Keywords:** Vocal Cord Paralysis, Phonology Impairment, Surgical Procedures, Operative

## Abstract

**Background:**

Revision laryngeal framework surgery is usually performed for medialisation laryngoplasty failure, rather than for failure after arytenoid adduction. We describe a new method for revision arytenoid adduction surgery, performed by directly pulling the lateral cricoarytenoid muscle (‘lateral cricoarytenoid muscle pull surgery’).

**Methods:**

We describe a case of revision laryngeal framework surgery, present a literature review and describe the advantages of lateral cricoarytenoid muscle pull surgery over the original method of arytenoid adduction using a posterior approach.

**Results:**

Medialisation laryngoplasty combined with arytenoid adduction was performed following unilateral vocal fold paralysis from mediastinal surgery, resulting in severe glottic insufficiency. The patient's voice improved after the initial surgery, but had deteriorated 18 months later. Revision surgery was performed using lateral cricoarytenoid muscle pull surgery, and her voice recovered normally in terms of perceptual impression. The post-operative course was uneventful for 10 months following revision surgery.

**Conclusion:**

To our knowledge, this is the first case of revision arytenoid adduction performed using a lateral cricoarytenoid muscle pull approach. Lateral cricoarytenoid muscle pull surgery should therefore be considered as a new fenestration approach for arytenoid adduction.

## Introduction

Unilateral vocal fold paralysis reduces a patient's quality of life by inducing severe dysphonia and aspiration. However, laryngeal framework surgery can significantly improve the symptoms of unilateral vocal fold paralysis. Laryngeal framework surgery comprises medialisation laryngoplasty and arytenoid adduction procedures. Since their introduction, these procedures have become common treatments for vocal fold paralysis and glottal incompetence. However, not all procedures provide satisfactory long-term phonological results. In some cases, symptoms have recurred, requiring revision surgery.^1^^,^^2^ In our experience, revision medialisation laryngoplasty is easy to perform, but revision arytenoid adduction is more difficult because the surgical field is severely scarred after primary arytenoid adduction. Several important anatomical structures such as the pyriform sinus mucosa and carotid artery can be affected. Thus, revision arytenoid adduction requires a different approach from primary arytenoid adduction.

A method involving directly pulling the lateral cricoarytenoid muscle was first reported by Iwamura and Kurita.^3^ Tokashiki and colleagues modified the procedure using a fenestration approach and obtained good phonological results in combination with medialisation laryngoplasty.^4^^,^^5^ This approach is simple and enables arytenoid adduction to be performed through a window made in the posterior lower thyroid alar cartilage to enable pulling of the lateral cricoarytenoid muscle or the muscle process of the arytenoid cartilage.^4^^,^^5^ The approach differs from the original Isshiki method, in which the thyroid alar cartilage is extended outward and the pyriform sinus mucosa is dissected to reach the cricoarytenoid joint (using a posterior approach).^6^ We recently performed medialisation laryngoplasty and arytenoid adduction using the lateral cricoarytenoid muscle pull method as revision surgery in a patient who had previously undergone medialisation laryngoplasty and arytenoid adduction using the original posterior approach.

## Case report

**Fig. 1:**
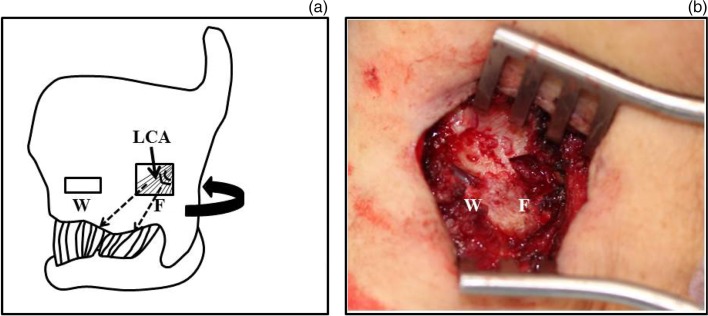
(a) Diagram showing differences between the lateral cricoarytenoid muscle (LCA) pull method and the original arytenoid adduction method using a posterior approach. The curved arrow indicates the direction of the original arytenoid adduction using the posterior approach. The dashed arrows represent sutures passed through the fenestration (F) to pull and fix the lateral cricoarytenoid muscle. The window (W) used for medialisation laryngoplasty is shown. The figure is modified from Tokashiki *et al*.^9^ (reprinted with permission). (b) Intra-operative image, showing a fenestration (F) in the upper rear of the medialisation laryngoplasty window (W) in the thyroid cartilage

A 69-year-old woman had breathy hoarseness and severe aspiration after mediastinal surgery to remove a metastatic breast cancer tumour. Laryngoscopy revealed that the left vocal fold was fixed in a lateral position, with vocal fold bowing. The maximum phonation time was 3 seconds and the mean flow rate was greater than 1000 ml/s. Shimmer or jitter could not be measured. Upon phonation, a wide posterior glottal chink was observed. Thus, arytenoid adduction combined with medialisation laryngoplasty (i.e. combined surgery) was required to improve these measures (Table I). First, combined surgery was performed in accordance with descriptions in previous reports.^7^ After creating a cervical incision, the thyroid cartilage was skeletonised. The inferior pharyngeal constrictor muscle was then removed from the thyroid cartilage to expose the posterior portion of the lamina. After forming a window for medialisation laryngoplasty, the thyroid cartilage was twisted and the surgical field was reached using a posterior approach (Figure 1a). The pyriform sinus mucosa was elevated from the underside of the thyroid cartilage to expose the muscular process of the arytenoid cartilage. The muscular process was then stitched with nylon suture and pulled to contract the lateral cricoarytenoid muscle. After arytenoid adduction, a strip of Gore-Tex (W. L. Gore, Flagstaff, Arizona, USA) was packed into the subperichondrial pocket of the medialisation laryngoplasty window. After the first surgery, phonological results were obviously improved. The maximum phonation time was 15 seconds and the mean flow rate was 149 ml/s. Shimmer and jitter values were 3.7 per cent and 0.4 per cent, respectively (Table I).

**Table I:** Voice evaluation before and after surgery

Parameter	First surgery		Second surgery	
	Before	After	Before	After
MPT (s)	3	15	4	11
MFR (ml/s)	>1000	149	766	173
Shimmer (%)	Aphonic	3.7	15.4	4.9
Jitter (%)	Aphonic	0.4	7.0	1.4

MPT = maximum phonation time; s = seconds; MFR = mean flow rate

**Fig. 2:**
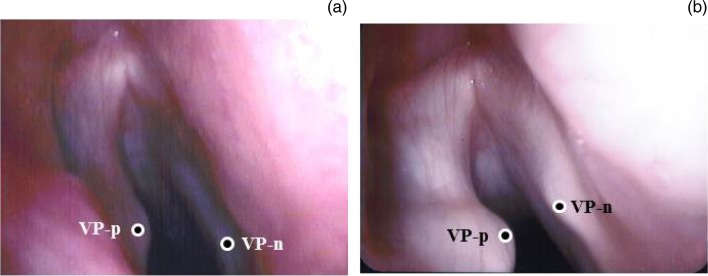
Positions of the paralysed side (VP-p) and normal side (VP-n) of the vocal process during surgery, as indicated by circles. (a) The VP-p was initially located at a higher point than the VP-n. (b) The VP-p was pulled down to a lower point than the VP-n using the lateral cricoarytenoid muscle pull method

However, 18 months after the primary surgery, the patient again experienced breathy hoarseness and aspiration, and the results of acoustic analysis were worse. The maximum phonation time decreased to 4 seconds and the mean flow rate increased to 766 ml/s. Shimmer and jitter values were 15.4 per cent and 7.0 per cent, respectively (Table I). Laryngoscopy revealed that the position of the Gore-Tex was unchanged; however, a posterior glottal chink was observed. Revision surgery was therefore performed to correct latent arytenoid adduction failure was thought to have occurred. The portion dissected in the first surgery was cicatrised, but it was difficult to identify the cricoarytenoid joint using a similar approach to the one used in the first surgery. Thus, to perform revision arytenoid adduction, a fenestration approach was used to pull the lateral cricoarytenoid muscle (Figure 1a). A fenestration was created at the upper rear of the medialisation laryngoplasty window in the thyroid cartilage (Figure 1b). Intra-operative laryngoscopy revealed that the paralysed side of the vocal process was located at a higher point than the normal side (Figure 2a). The lateral cricoarytenoid muscle was identified through the fenestration and pulled anteriorly using a 4-0 gauge nylon thread. The paralysed side of the vocal process was pulled down to a lower point than the normal side of the vocal process using the lateral cricoarytenoid muscle pull method (Figure 2b). The adductive effect of the lateral cricoarytenoid muscle pull method was similar to that of the original arytenoid adduction, as previously reported.^7^ Medialisation laryngoplasty was then performed in the same way as the primary operation. The maximum phonation time improved to 11 seconds, and the mean flow rate decreased to 173 ml/s. Shimmer and jitter values were 4.9 per cent and 1.4 per cent, respectively, after the operation. These values were all within the normal ranges, and the patient's voice recovered to normal in terms of perceptual impression. Her post-operative course was uneventful for 10 months after the revision surgery.

### Ethical standards

All procedures were performed in compliance with the ethical standards of the relevant national and institutional guidelines on human experimentation (Shinn-Oyama City Hospital) and with the Helsinki Declaration of 1975, as revised in 2008.

## Discussion

Laryngeal framework surgery is a well-known surgical procedure for treating unilateral vocal fold paralysis. Medialisation laryngoplasty and/or arytenoid adduction can be performed, depending on the severity of hoarseness. In the case of severe unilateral vocal fold paralysis, arytenoid adduction combined with medialisation laryngoplasty (i.e. combined surgery) is needed to obtain acceptable results.^8^ Although good vocal outcomes after laryngeal framework surgery have been described, they can be variable and may change over time. When adequate phonological results are not achieved, a revision procedure may be indicated. Young *et al*. described revision surgery following laryngeal framework surgery in 6 per cent of cases.^1^ Common revision surgeries involved replacement with a larger (in 37 per cent of all medialisation laryngoplasty revisions) or smaller (in 8 per cent of cases) implant, and repositioning of the implant (in 24 per cent of cases).^1^ Arytenoid adduction and vocal fold injection augmentation were performed in 10.3 per cent and 19.7 per cent of surgeries, respectively.^1^ However, there are no reports of revision surgery for arytenoid adduction itself. This may be because arytenoid adduction is a stable surgical procedure, with little chance of failure. Alternatively, the surgical field following primary arytenoid adduction may contain severe scarring that might cause pyriform sinus mucosal damage, leading to difficulties in revision surgery.

We directly pulled the lateral cricoarytenoid muscle using a fenestration approach and observed a good phonological outcome. The fenestration approach was first proposed by Iwamura and Kurita^3^ in the Japanese literature and was later modified by Tokashiki *et al*.^4^^,^^5^ Of course, this procedure is also useful as a primary surgery, and its advantages in this context have been reported.^4^^,^^5^^,^^9^^,^^10^ Both fenestration and posterior approaches can be used to perform arytenoid adduction and medialisation laryngoplasty procedures.^4^^,^^11^ The difference between these methods is that the fenestration approach does not remove the posterior border of the thyroid cartilage. Maragos reported that 6.8 per cent of his patients receiving arytenoid adduction by a posterior approach needed post-operative tracheostomy, and that airway narrowing induced by removal of the posterior portion caused airway obstruction.^12^ To avoid this complication, he recommended stabilising the elevated pyriform sinus mucosa to the thyroid cartilage.^13^ He considered preservation of the posterior portion of the thyroid cartilage, as in the fenestration approach, to be useful because the pyriform attaches to the cartilage. Another difference is the direction of suture pulling in arytenoid adduction. The simultaneous performance of arytenoid adduction and medialisation laryngoplasty procedures has been reported. In all reports, the suture was fixed to the anterior–inferior part of the thyroid cartilage, as in Isshiki and colleague's original approach.^6^ In these procedures, the suture runs across medialisation laryngoplasty window, and may therefore interfere with medialisation laryngoplasty.^4^^,^^5^ In the present procedure, the suture was pulled in the contractile direction of the lateral cricoarytenoid muscle and fixed it to the lower edge of the thyroid cartilage. This procedure has the advantages of not interrupting the medialisation laryngoplasty surgical field and of reproducing the natural adduction of the arytenoid cartilage.^4^ The lateral cricoarytenoid muscle plays the most important role in vocal fold adduction. Su *et al*. demonstrated that arytenoid adduction with a suture attachment to the cricoid cartilage along the longitudinal axis of the lateral cricoarytenoid muscle is more physiological and effective than suture attachment to the anterolateral part of the thyroid ala.^4^^,^^13^

• Poor vocal outcomes after laryngeal framework surgery may require revision surgery
• A good phonological outcome was observed after directly pulling the lateral cricoarytenoid muscle using a fenestration approach
• This approach has several advantages for both primary and revision surgery

Furthermore, laryngeal framework surgery is usually performed under local anaesthesia, but some patients require general anaesthesia because of their poor physical condition. A laryngeal mask airway device is a good tool for laryngeal framework surgery under general anaesthesia.^7^^,^^9^ However, the pyriform sinus mucosa is extended outward from the bulge caused by the device, thus making it difficult to expose the muscular process using the posterior approach. The fenestration approach avoids this problem, and makes it convenient to perform adduction under general anaesthesia using a laryngeal mask airway device.^9^

Although it is unclear why a thread from the arytenoid adduction had loosened, this problem was successfully resolved following revision arytenoid adduction surgery using a fenestration approach. To the best of our knowledge, this is the first case report of revision arytenoid adduction performed using the lateral cricoarytenoid muscle pull method. This method has several advantages not only for primary surgery but also for revision surgery, and deserves consideration as a new fenestration approach.
